# TWEAK/Fn14 interaction regulates RANTES production, BMP-2-induced differentiation, and RANKL expression in mouse osteoblastic MC3T3-E1 cells

**DOI:** 10.1186/ar2038

**Published:** 2006-09-01

**Authors:** Takashi Ando, Jiro Ichikawa, Masanori Wako, Kyosuke Hatsushika, Yoshiyuki Watanabe, Michitomo Sakuma, Kachio Tasaka, Hideoki Ogawa, Yoshiki Hamada, Hideo Yagita, Atsuhito Nakao

**Affiliations:** 1 Department of Immunology, Faculty of Medicine, University of Yamanashi, 1110 Shimokato, Chuo, Yamanashi 409-3898, Japan; 2 Department of Orthopedic Surgery, Faculty of Medicine, University of Yamanashi, 1110 Shimokato, Chuo, Yamanashi 409-3898, Japan; 3 Atopy Research Center, Juntendo University School of Medicine, 2-1-1, Hongo, Bunkyo-ku, Tokyo 113-8421, Japan; 4 Department of Immunology, Juntendo University School of Medicine, 2-1-1, Hongo, Bunkyo-ku, Tokyo 113-8421, Japan

## Abstract

Tumour necrosis factor (TNF)-like weak inducer of apoptosis (TWEAK), a member of the TNF family, is a multifunctional cytokine that regulates cell growth, migration, and survival principally through a TWEAK receptor, fibroblast growth factor-inducible 14 (Fn14). However, its physiological roles in bone are largely unknown. We herein report various effects of TWEAK on mouse osteoblastic MC3T3-E1 cells. MC3T3-E1 cells expressed Fn14 and produced RANTES (regulated upon activation, healthy T cell expressed and secreted) upon TWEAK stimulation through PI3K-Akt, but not nuclear factor-κB (NF-κB), pathway. In addition, TWEAK inhibited bone morphogenetic protein (BMP)-2-induced expression of osteoblast differentiation markers such as alkaline phosphatase through mitogen-activated protein kinase (MAPK) Erk pathway. Furthermore, TWEAK upregulated RANKL (receptor activation of NF-κB ligand) expression through MAPK Erk pathway in MC3T3-E1 cells. All these effects of TWEAK on MC3T3-E1 cells were abolished by mouse Fn14-Fc chimera. We also found significant TWEAK mRNA or protein expression in osteoblast – and osteoclast-lineage cell lines or the mouse bone tissue, respectively. Finally, we showed that human osteoblasts expressed Fn14 and induced RANTES and RANKL upon TWEAK stimulation. Collectively, TWEAK/Fn14 interaction regulates RANTES production, BMP-2-induced differentiation, and RANKL expression in MC3T3-E1 cells. TWEAK may thus be a novel cytokine that regulates several aspects of osteoblast function.

## Introduction

Tumour necrosis factor (TNF)-like weak inducer of apoptosis (TWEAK) is a type II transmembrane protein belonging to the TNF superfamily and can also function as a secreted cytokine like TNF-α [1]. The soluble form of TWEAK has been shown to exert multiple biological activities, including stimulation of cell growth, angiogenesis, induction of inflammatory cytokines, and stimulation of apoptosis, principally through a TWEAK receptor named fibroblast growth factor-inducible 14 (Fn14) [2,3]. Fn14 is a type I transmembrane protein composed of only one cysteine-rich domain in the extra-cellular region and a short cytoplasmic region containing a TNF receptor-associated factor (TRAF)-binding motif [4,5]. TWEAK/Fn14 interaction stimulates canonical and non-canonical nuclear factor-κB (NF-κB) signaling pathways mediated by IκBα phosphorylation and p100 processing via TRAF molecules [6] and also stimulates mitogen-activated protein kinases (MAPKs) JNK (c-Jun NH_2_-terminal kinase), p38, and Erk [7].

We and others have shown that TWEAK/Fn14 interaction plays important roles in proliferation, migration, inflammatory responses, and survival in a variety of cell types, including endothelial, epithelial, immune, and some tumour cells [2,3,8-11]. In bone, Polek *et al*. reported that TWEAK induced differentiation of RAW264 monocyte/macrophage cells into osteoclasts in an Fn14-independent manner [12]. However, the effects of TWEAK on osteoblastic cells and its interplay with other cytokines still remain largely unknown.

In the present study, we thus investigated the biological effects of TWEAK on mouse MC3T3-E1 cells, a clonal osteogenic cell line that maintains characteristics of primary osteoblast progenitors and is frequently used for studying osteoblast differentiation and function *in vitro *[13]. Our results suggest that TWEAK/Fn14 interaction may regulate osteoblast functions.

## Materials and methods

### Reagents

Recombinant mouse TWEAK, human bone morphogenetic protein (BMP)-2, mouse TNF-α, and mouse Fn14-Fc chimera were purchased from R&D Systems Inc. (Minneapolis, MN, USA). Anti-Fn14 monoclonal antibody (mAb) (ITEM-1) was generated in our laboratory as previously described [9]. PD98059 and LY294002 were purchased from Calbiochem (San Diego, CA, USA), and Helenalin was purchased from BIOMOL International, L.P. (Plymouth Meeting, PA, USA).

### Cell culture

MC3T3-E1, RAW264, ATDC5, and EL4 cells were purchased from Riken Cell Bank (Tsukuba, Japan). MC3T3-E1 cells were maintained in α-minimal essential medium (Invitrogen, Carlsbad, CA, USA) with L-glutamine supplemented with 10% fetal calf serum, 100 μg/ml streptomycin, and 100 units per ml penicillin G solution. Primary osteoblasts derived from healthy human femoral bone from a patient with rheumatoid arthritis were purchased from Cell Applications, Inc. (San Diego, CA, USA) and were maintained using the manufacturer's recommended growth medium and supplements.

### Flow cytometric analysis

Cells (1 × 10^6^) were incubated with anti-Fn14 mAb (ITEM-1) for 1 hour at 4°C, followed by phycoerythrin-labeled rabbit anti-mouse immunoglobulin G (IgG) antibody (BD Pharmingen, San Diego, CA, USA). After a washing with phosphate-buffered saline (PBS), the cells were analyzed on a FACSCalibur (BD Biosciences, San Jose, CA, USA), and the data were analyzed with the WinMDI program (The Scripps Research Institute, La Jolla, CA, USA).

### Immunoblotting

Immunoblotting was performed as previously described [14] with specific antibodies for phosphorylated (Ser536)-NF-κB p65 (Cell Signaling Technology, Inc., Danvers, MA, USA), phosphorylated-Akt and Akt (Becton, Dickinson and Company, Franklin Lakes, NJ, USA), and phosphorylated-Erk p42/44 and Erk p42/44 (Cell Signaling Technology, Inc.).

### Cytokine protein array

Cells (1 × 10^6^) were stimulated with 100 ng/ml TWEAK for 48 hours. The amounts of several cytokines in the culture supernatants were then determined by using a cytokine protein array (TranSignal Mouse Cytokine Antibody Array 1.0; Panomics, Inc., Fremont, CA, USA) according to the manufacturer's instructions.

### Enzyme-linked immunosorbent assay

Cells (1 × 10^6^) were stimulated with 100 ng/ml TWEAK in the presence or absence of the indicated inhibitors for 48 hours. The amounts of RANTES (regulated upon activation, healthy T cell expressed and secreted) in the culture supernatants were then determined by using the mouse RANTES enzyme-linked immunosorbent assay (ELISA) kit (R&D Systems Inc.) according to the manufacturer's instructions.

### Cell viability assay

Cells (5 × 10^3^) were cultured with or without 100 ng/ml TWEAK in the presence or absence of the indicated inhibitors in a flat-bottom 96-well microtiter plate. The number of viable cells in each well was then determined by the WST (water-soluble tetrazolium) assay with the Tetra Color ONE kit (Seikagaku Corporation, Tokyo, Japan) according to the manufacturer's instructions.

### Alkaline phosphatase assay

Cells (5 × 10^3^/well) were cultured with 100 ng/ml BMP-2 for 5 days in the presence or absence of the indicated inhibitors in a flat-bottom 96-well plate. Alkaline phosphatase (ALP) activity was determined by the TRACP & ALP double-stain kit (Takara Bio Inc., Shiga, Japan).

### Reverse transcription-polymerase chain reaction

Reverse transcription-polymerase chain reaction (PCR) was performed as previously described [14]. PCR amplification (osteocalcin: 94°C for 0.5 minutes, 58°C for 0.5 minutes, and 72°C for 1 minute [35 cycles]; β-actin: 94°C for 0.5 minutes, 56°C for 0.5 minutes, and 72°C for 1 minute [35 cycles]) was performed in a DNA engine cycler (MJ Research, Inc., Watertown, MA, USA). The PCR products were separated by 2.0% agarose gel electrophoresis and stained with 0.5 μg/ml ethidium bromide. The primers used were mouse osteocalcin (forward, 5'-GCAGCTTGGTGCACACCTAG-3'; reverse, 5'-GGAGCTGCTGTGACATCCAT-3') and mouse β-actin (forward, 5'-TGGAATCCTGTGGCATCCATGAAAC-3'; reverse, 5'-TAAAACGCAGCTCAGTAACAGTCCG-3').

### Quantitative real-time PCR

Total RNA was extracted from cells (1 × 10^6^) or Balb/c mouse tissues. cDNA was then synthesised from 2 μg of total RNA by using the Reverse Transcriptase System (Applied Biosystems, Foster City, CA, USA). Quantitative PCR analysis was performed using the ABI7500 real-time PCR system (Applied Biosystems) according to the manufacturer's instructions. Primers and probes for mouse RANKL (receptor activation of nuclear factor-κB ligand), mouse TWEAK, and glyceraldehyde-3-phosphate dehydrogenase (GAPDH) were purchased from Applied Biosystems. The ratio of each gene to that of GAPDH was calculated, and the value of 1.0 was assigned to cells that were incubated without transforming growth factor (TGF)-β1 or TWEAK.

### Immunofluorescence study

Cells were grown on eight-well culture slides with 100 ng/ml TWEAK for 24 or 48 hours, washed with PBS, and fixed with 4% paraformaldehyde. After permeabilisation, slides were stained with goat polyclonal anti-RANKL antibody or control goat IgG (Santa Cruz Biotechnology, Inc., Santa Cruz, CA, USA) diluted in PBS overnight at 4°C. After extensive washing, slides were incubated with rabbit polyclonal fluorescein isothiocyanate-conjugated anti-goat antibody (1:200) (DakoCytomation, Glostrup, Denmark) for 1 hour at room temperature. Images were acquired with a confocal microscopy (ECLIPSE E800; Nikon Corporation, Tokyo, Japan).

### Immunohistochemistry

Mouse tail bones obtained from 4- to 6-week-old female Balb/c mice were dissected, fixed, decalcified, and embedded in paraffin according to conventional methods. Sections were then stained with goat anti-TWEAK antibody (Santa Cruz Biotechnology, Inc.) or control goat IgG (Santa Cruz Biotechnology, Inc.) through the use of the peroxidase-based VECTASTAIN ABC kits with DAB (diaminobenzidine) substrate (Vector Laboratories, Burlingame, CA, USA).

### Data analysis

Data are represented as the mean ± standard deviation of triplicate samples. Statistical analysis was performed by the unpaired Student's *t *test. *P *< 0.05 was considered to be significant.

## Results

### MC3T3-E1 cells express functional Fn14

**Figure 1:**
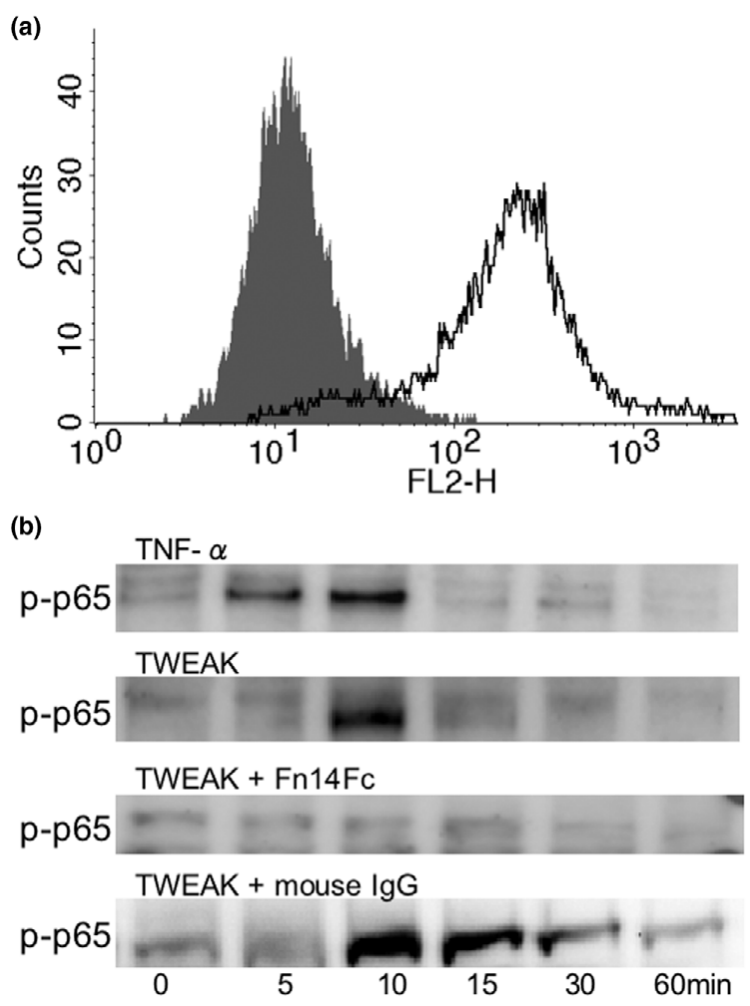
MC3T3-E1 cells express functional Fn14. **(a) **Cell surface expression of Fn14 on MC3T3-E1 cells. MC3T3-E1 cells were stained with anti-Fn14 monoclonal antibody (open histogram) or control Ig (filled histogram) followed by phycoerythrin-labeled rabbit anti-mouse Ig antibody and analyzed by flow cytometry. **(b) **TWEAK phosphorylates the NF-κB p65 subunit in MC3T3-E1 cells. MC3T3-E1 cells were stimulated with 100 ng/ml TWEAK or 10 ng/ml TNF-α (as a positive control) in the presence or absence of 1 μg/ml mouse Fn14-Fc chimera (Fn14-Fc) or control mouse IgG for the indicated time periods. The cell lysates were then subjected to immunoblotting with antibody specific for the Ser536-phosphorylated NF-κB p65 subunit. Fn14, fibroblast growth factor-inducible 14; Ig, immunoglobulin; NF-κB, nuclear factor-κB; TNF-α, tumour necrosis factor-α; TWEAK, tumour necrosis factor-like weak inducer of apoptosis.

To investigate the effects of TWEAK on osteoblastic cells, we first examined whether a functional TWEAK receptor, Fn14, was expressed on MC3T3-E1 cells. As shown in Figure 1a, fluorescence-activated cell sorting analysis with anti-Fn14 mAb showed that Fn14 was significantly expressed on the surface of MC3T3-E1 cells. Moreover, TWEAK induced rapid phosphorylation of the NF-κB p65 subunit in MC3T3-E1 cells, which was abrogated by the addition of mouse Fn14-Fc chimera (Figure 1b).

### TWEAK/Fn14 interaction induces RANTES production by MC3T3-E1cells

**Figure 2:**
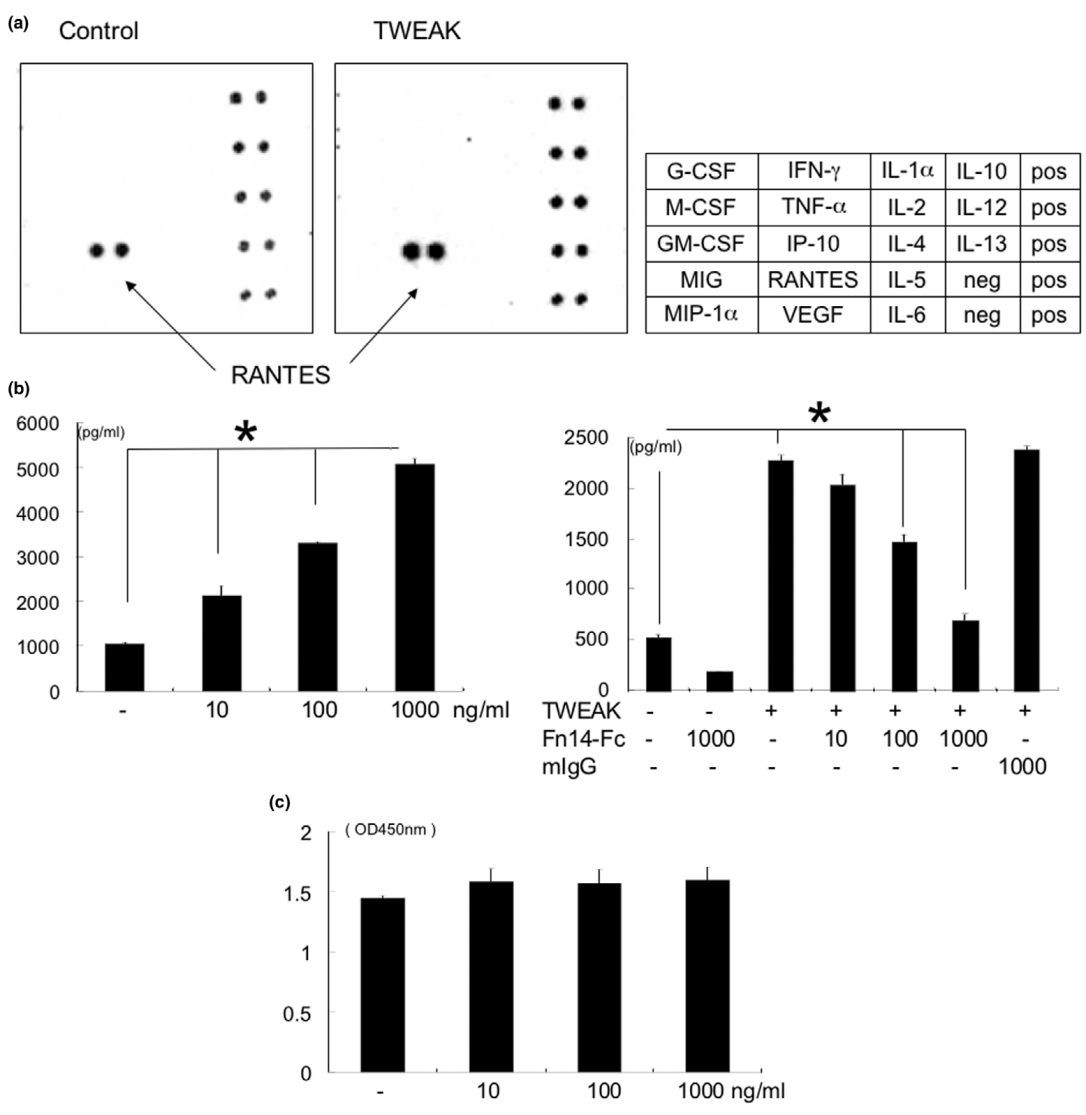
TWEAK/Fn14 interaction induces RANTES production by MC3T3-E1 cells. **(a) **MC3T3-E1 cells were cultured in the presence or absence of 100 ng/ml TWEAK for 48 hours. The culture supernatants were then collected and subjected to a cytokine protein array. The table indicates the corresponding cytokines on the protein array membrane. **(b) **MC3T3-E1 cells were stimulated with the indicated doses of TWEAK for 48 hours in the presence or absence of the indicated doses (ng/ml) of mouse Fn14-Fc chimera or control mouse immunoglobulin G (mIgG). The culture supernatants were then collected, and the RANTES concentrations were measured by enzyme-linked immunosorbent assay. **(c) **MC3T3-E1 cells were stimulated with the indicated doses of TWEAK for 96 hours. Viable cell number was then measured by WST assay. The data were expressed as OD units. Values represent the mean ± standard deviation. *p < 0.05 compared with corresponding control. Similar results were obtained in at least three independent experiments. Fn14, fibroblast growth factor-inducible 14; G-CSF, granulocyte-cell-stimulating factor; GM-CSF, granulocyte macrophage-colony-stimulating factor; IFN-γ, interferon-γ; IL, interleukin; IP-10, inositol phosphate-10; M-CSF, macrophage-colony-stimulating factor; MIG, monokine induced by IFN-γ; MIP-1α, viral macrophage inflammatory protein-1α; OD, optical density; RANTES, regulated upon activation, healthy T cell expressed and secreted; TNF-α, tumour necrosis factor-α; TWEAK, tumour necrosis factor-like weak inducer of apoptosis; VEGF, vascular endothelial growth factor; WST, water-soluble tetrazolium.

Because MC3T3-E1 cells expressed Fn14, we next examined the effects of TWEAK on cytokine production by MC3T3-E1 cells by using a cytokine protein array. We found that TWEAK exclusively induced RANTES in this assay (Figure 2a). Using an ELISA, we confirmed that TWEAK significantly induced RANTES production by MC3T3-E1 cells in a dose-dependent manner and that the blockade of Fn14 with Fn14-Fc chimera inhibited TWEAK-induced RANTES production (Figure 2b). TWEAK did not affect cellular viability at the doses used in the experiments (Figure 2c).

### TWEAK-induced RANTES production by MC3T3-E1 cells involves the PI3K-Akt pathway

**Figure 3:**
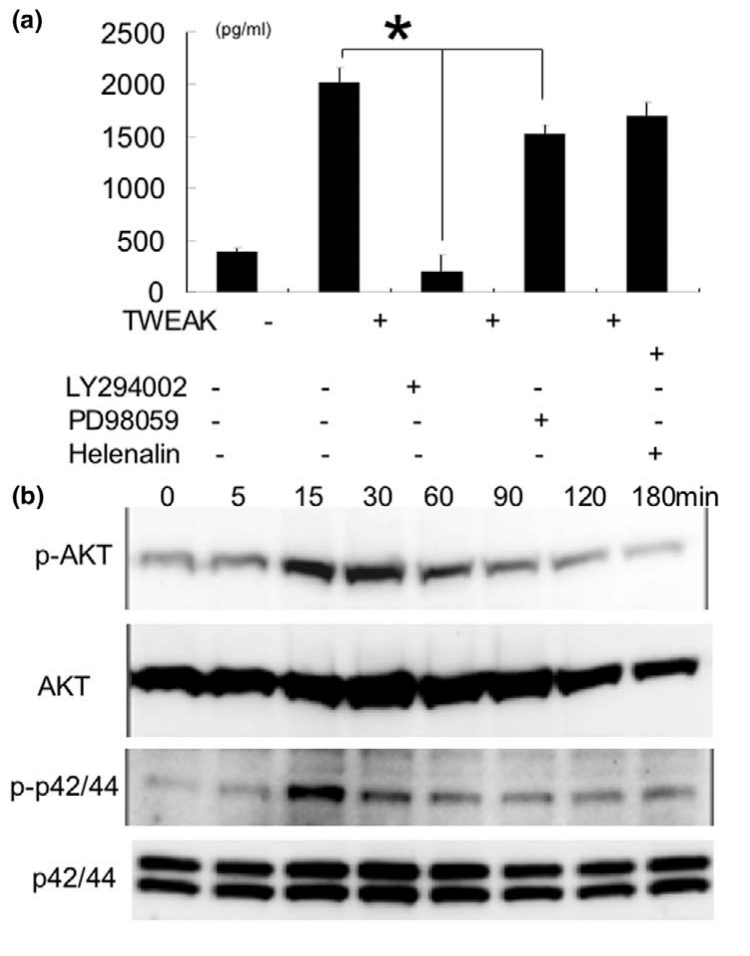
TWEAK-induced RANTES production by MC3T3-E1 cells involves the PI3K-Akt pathway. **(a) **MC3T3-E1 cells were cultured in the presence or absence of 100 ng/ml TWEAK with or without 10 μM LY294002, 10 μM PD98059, or 3 μM Helenalin for 48 hours. The culture supernatants were then collected, and the RANTES concentrations were measured by enzyme-linked immunosorbent assay. **(b) **MC3T3-E1 cells were stimulated with 100 ng/ml TWEAK for the indicated time periods. The cell lysates were then subjected to immunoblotting with antibody specific for phosphorylated Akt, Akt, phosphorylated Erk p42/44, and Erk p42/44. Values represent the mean ± standard deviation. *p < 0.05 compared with corresponding control. Similar results were obtained in at least three independent experiments. RANTES, regulated upon activation, healthy T cell expressed and secreted; TWEAK, tumour necrosis factor-like weak inducer of apoptosis.

To clarify intracellular signaling pathways involved in TWEAK-induced RANTES production in MC3T3-E1 cells, we examined the effects of several inhibitors on TWEAK-induced RANTES production in MC3T3-E1 cells. Helenalin (an inhibitor of NF-κB) did not affect the TWEAK-induced RANTES production by MC3T3-E1 cells, and PD98059 (an inhibitor of the MAPK Erk pathway) marginally affected the TWEAK-induced RANTES production by MC3T3-E1 cells (Figure 3a). However, LY294002, an inhibitor of PI3K, significantly suppressed TWEAK-induced RANTES production (Figure 3a). Consistent with these findings, TWEAK induced phosphorylation of Akt, a downstream target of PI3K (Figure 3b). Phosphorylation of NF-κB subunit p65 and Erk p42/44 was also induced by TWEAK in MC3T3-E1 cells (Figures 1b and 3b). These results indicated that TWEAK activates several intracellular signaling pathways in MC3T3-E1 cells but that the PI3K-Akt pathway is involved in TWEAK-induced RANTES production in MC3T3-E1 cells.

### TWEAK inhibits BMP-induced osteoblast differentiation ofMC3T3-E1 cells

**Figure 4:**
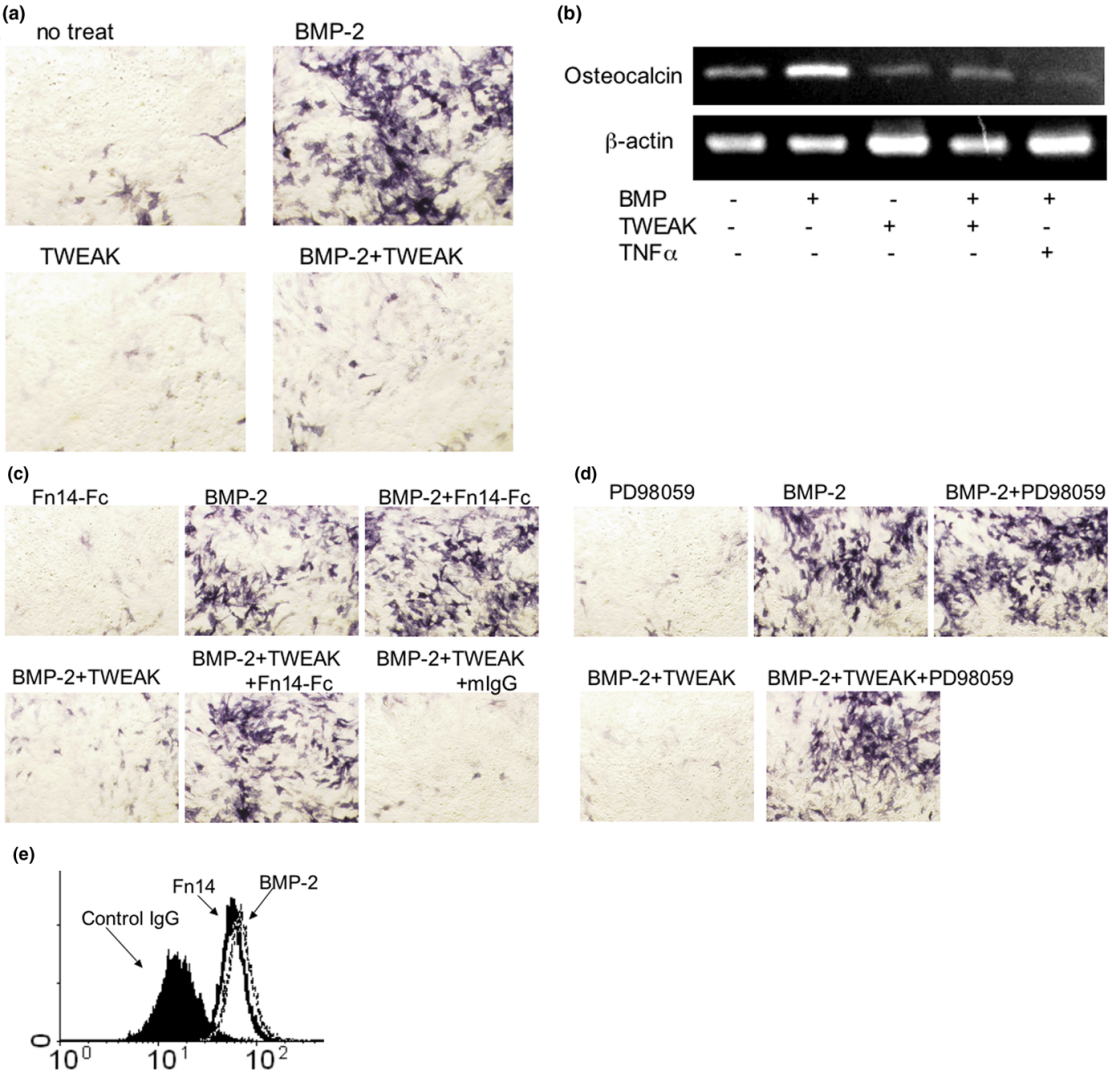
TWEAK inhibits BMP2-induced osteoblastic differentiation in MC3T3-E1 cells. **(a) **MC3T3-E1 cells were stimulated with 100 ng/ml BMP-2 in the presence or absence of 100 ng/ml TWEAK for 5 days, and then ALP activity was determined using the TRACP & ALP double-stain kit. TWEAK inhibited BMP-2-induced ALP activity. Representative images are shown. **(b) **MC3T3-E1 cells were stimulated with 100 ng/ml BMP-2 with or without 100 ng/ml TWEAK or with 10 ng/ml TNF-α (as a positive control) for 48 hours. Osteocalcin mRNA expression was then evaluated by reverse transcription-polymerase chain reaction. **(c) **MC3T3-E1 cells were stimulated with 100 ng/ml BMP-2 in the presence or absence of 100 ng/ml TWEAK and/or 1 μg/ml Fn14-Fc chimera (Fn14-Fc) or control mouse IgG (mIgG) for 5 days, and then ALP activity was determined using the TRACP & ALP double-stain kit. Fn14-Fc chimera abrogated TWEAK inhibition of BMP-2-induced ALP activity. Representative images are shown. **(d) **MC3T3-E1 cells were stimulated with 100 ng/ml BMP-2 in the presence or absence of 100 ng/ml TWEAK and/or 1 μM PD98059 for 5 days, and then ALP activity was determined using the TRACP & ALP double-stain kit. PD98059 abrogated TWEAK inhibition of BMP-2-induced ALP activity. Representative images are shown. **(e) **MC3T3-E1 cells were cultured in the presence or absence of 100 ng/ml BMP-2 for 24 hours. The cells were then stained with anti-Fn14 monoclonal antibody (open histogram, unbroken line) or control Ig (filled histogram) followed by phycoerythrin-labeled rabbit anti-mouse Ig antibody and were analyzed by flow cytometry. Addition of BMP-2 did not affect surface Fn14 expression (open histogram, dotted line). Similar results were obtained in at least three independent experiments. ALP, alkaline phosphatase; BMP-2, bone morphogenetic protein-2; Fn14, fibroblast growth factor-inducible 14; Ig, immunoglobulin; TNF-α, tumour necrosis factor-α; TWEAK, tumour necrosis factor-like weak inducer of apoptosis.

BMPs are important members of the TGF-β superfamily and control osteogenesis [15,16], and BMP-2 can induce differentiation of preosteoblastic MC3T3-E1 cells into osteoblastic phenotype as demonstrated by increased ALP activity and osteocalcin expression [17]. We then investigated whether TWEAK affected BMP-2-induced differentiation of MC3T3-E1 cells. MC3T3-E1 cells cultured for 5 days in the presence of BMP-2 significantly developed ALP activity, which was inhibited by the addition of TWEAK (Figure 4a). Osteocalcin mRNA expression induced by BMP-2 was also inhibited by TWEAK (Figure 4b). The inhibitory effect of TWEAK on BMP-2-induced ALP activity in MC3T3-E1 cells was abrogated by the addition of Fn14-Fc chimera (Figure 4c). Furthermore, the inhibitory effect of TWEAK on BMP-2-induced ALP activity in MC3T3-E1 cells was also abrogated by the addition of PD98059 (Figure 4d). We confirmed that BMP-2 did not affect cell surface Fn14 expression on MC3T3-E1 cells (Figure 4e). These results indicate that TWEAK inhibited BMP-2-induced osteoblastic differentiation in MC3T3-E1 cells through the Fn14 and MAPK Erk pathways.

### TWEAK induces RANKL expression in MC3T3-E1 cells

**Figure 5:**
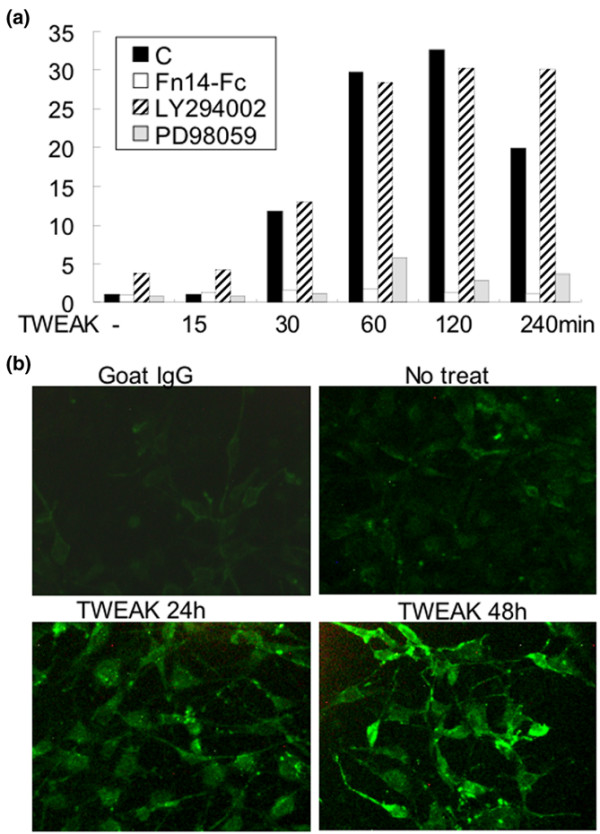
TWEAK/Fn14 interaction induces RANKL expression in MC3T3-E1 cells. **(a) **MC3T3-E1 cells were stimulated with 100 ng/ml TWEAK for the indicated times in the absence or presence of 1 μg/ml Fn14-Fc chimera, 10 μM LY2940002, or 10 μM PD98059. RNA was then extracted from the cells, and real-time PCR was performed using specific primers for RANKL and GAPDH (C, no treatment). The ratio of each gene to that of GAPDH was calculated, and the value of 1.0 was assigned to MC3T3-E1 cells that were incubated without TWEAK. **(b) **MC3T3-E1 cells were grown on slide chambers, stimulated with 100 ng/ml TWEAK for 24 and 48 hours, and then stained for RANKL as described in Materials and methods. RANKL-positive cells are shown in green. Goat IgG antibody was used as a negative control for the immunofluorescence staining. Fn14, fibroblast growth factor-inducible 14; GAPDH, glyceraldehyde-3-phosphate dehydrogenase; IgG, immunoglobulin G; RANKL, receptor activation of nuclear factor-κB ligand; TWEAK, tumour necrosis factor-like weak inducer of apoptosis.

RANKL is an important ligand expressed on osteoblasts for osteoclast differentiation [18]. We then investigated whether TWEAK affected RANKL expression in MC3T3-E1 cells. TWEAK-induced RANKL mRNA expression in MC3T3-E1 cells peaked at 120 minutes after the stimulation and was inhibited by Fn14-Fc chimera (Figure 5a). TWEAK-induced RANKL mRNA expression was also abrogated by PD98059, but not LY294002 (Figure 5a). An immunofluorescence study also showed that TWEAK induced RANKL expression at the protein level (Figure 5b). These results indicate that TWEAK induced RANKL expression in MC3T3-E1 cells through the Fn14 and MAPK Erk pathways.

### MC3T3-E1 and RAW264 cells express TWEAK mRNA

**Figure 6:**
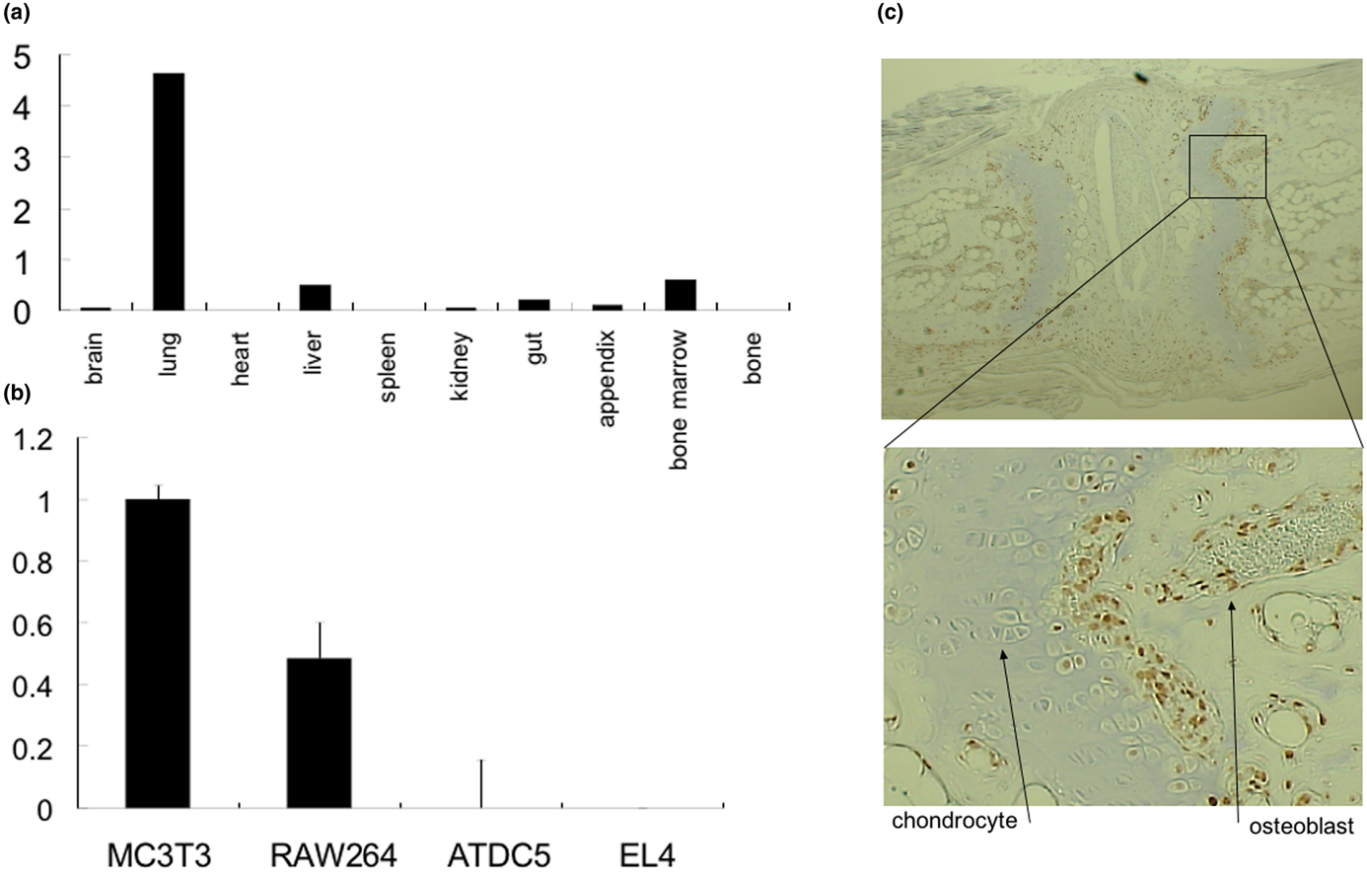
TWEAK expression in bone-related cell lines and the mouse bone tissue. **(a) **The indicated tissues were obtained from Balb/c mice, and RNA was extracted. Real-time PCR was then performed using specific primers for TWEAK and GAPDH. The ratio of each gene to that of GAPDH was calculated, and the value of 0.1 was assigned to the brain tissue. **(b) **RNA was extracted from MC3T3-E1, RAW264, ATDC5, and EL4 cells, and then real-time PCR was performed using specific primers for TWEAK and GAPDH. The ratio of each gene to that of GAPDH was calculated, and the value of 0.1 was assigned to MC3T3-E1 cells. **(c) **Immunohistochemical examination of the mouse bone tissue. Mouse tail bone tissues from Balb/c mice were stained with anti-TWEAK antibody. Representative images of weak enlargement (upper panel) and strong enlargement (lower panel) are shown. Positive staining is indicated as brown. GAPDH, glyceraldehyde-3-phosphate dehydrogenase; PCR, polymerase chain reaction; TWEAK, tumour necrosis factor-like weak inducer of apoptosis.

Because we found that TWEAK affected various osteoblast functions, we then investigated whether TWEAK mRNA was expressed in various mouse tissues and mouse bone cell lines. We found that TWEAK mRNA expression was relatively high in the lung, liver, and bone marrow (Figure 6a). We also found that TWEAK mRNA was expressed in MC3T3-E1 cells and RAW264 cells, a mouse monocyte/osteoclast cell line (Figure 6b). Mouse chondrocyte cell line ATDC5 and mouse thymic tumour cell line EL4 did not express TWEAK mRNA (Figure 6b). Consistent with these findings, an immunohistochemical analysis with anti-TWEAK antibody showed that TWEAK immunoreactivity was detected in morphologically osteoblast- or osteocyte-like cells, but not in chondrocytes, in the mouse bone tissue (Figure 6c). Staining with control IgG did not show any immunoreactivity in the bone tissue (data not shown).

### Human osteoblasts express Fn14 and induce RANTES and RANKL upon TWEAK stimulation

**Figure 7:**
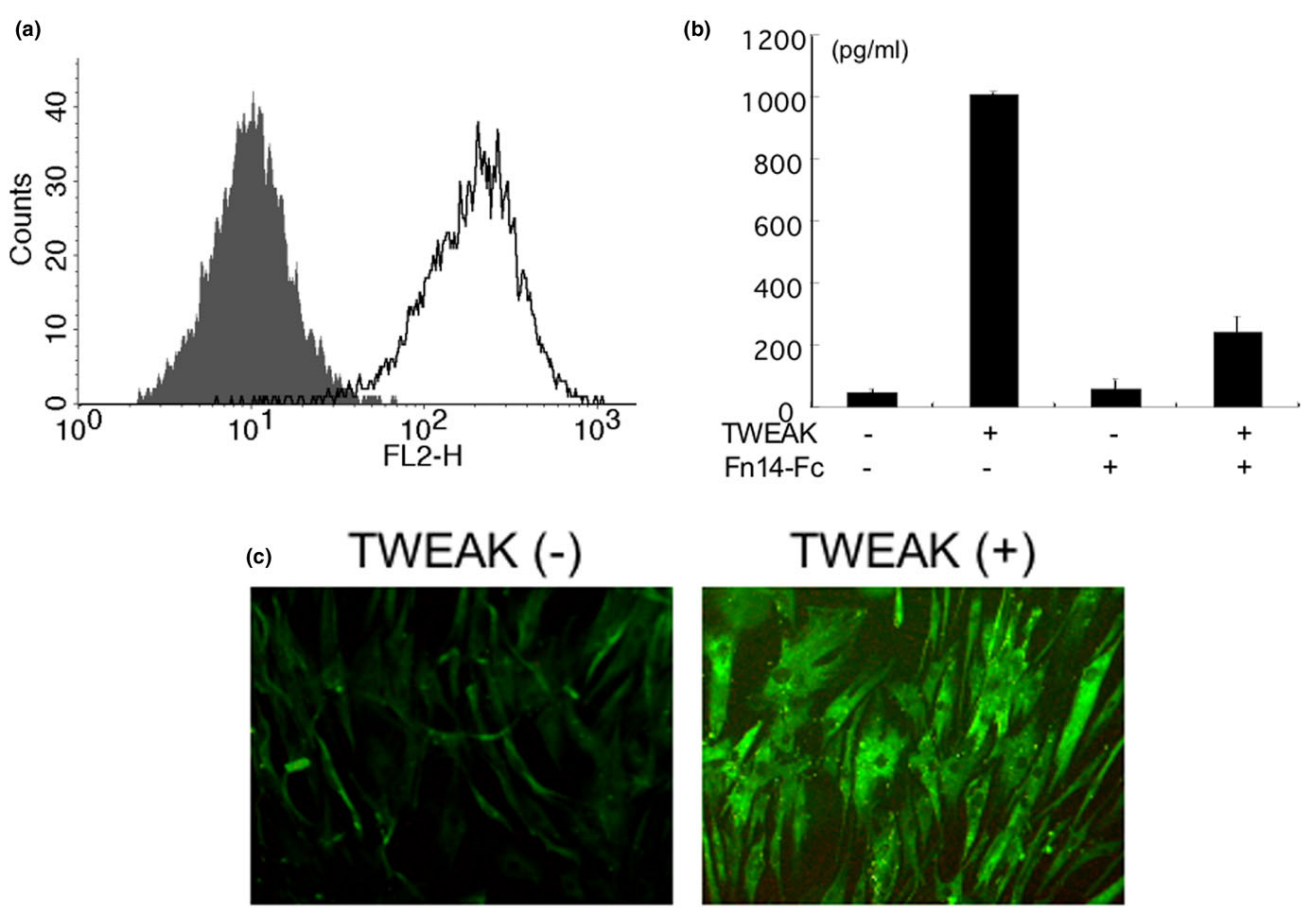
Human primary osteoblasts express Fn14 and induce RANTES and RANKL upon TWEAK stimulation. **(a) **Cell surface expression of Fn14 on human osteoblasts. Human osteoblasts were stained with anti-Fn14 monoclonal antibody (open histogram) or control Ig (filled histogram) followed by phycoerythrin-labeled rabbit anti-mouse Ig antibody and were analyzed by flow cytometry. **(b) **Human osteoblasts were stimulated with 100 ng/ml TWEAK for 48 hours in the absence or presence of 1 μg/ml Fn14-Fc chimera (Fn14-Fc). The culture supernatants were then collected, and the RANTES concentrations were measured by enzyme-linked immunosorbent assay. Values represent the mean ± standard deviation. *p < 0.05 compared with corresponding control. **(c) **Human osteoblasts were grown on slide chambers, stimulated with 100 ng/ml TWEAK for 48 hours, and then stained for RANKL as described in Materials and methods. RANKL-positive cells are shown in green. Goat IgG antibody was used as a negative control for the immunofluorescence staining, with negative results (data not shown). BMP, bone morphogenetic protein; Fn14, fibroblast growth factor-inducible 14; Ig, immunoglobulin; RANKL, receptor activation of nuclear factor-κB ligand; RANTES, regulated upon activation, healthy T cell expressed and secreted; TWEAK, tumour necrosis factor-like weak inducer of apoptosis.

Finally, we investigated whether TWEAK had some effects on primary human osteoblasts. As shown in Figure 7a, cultured osteoblasts derived from healthy human femoral bone from a patient with rheumatoid arthritis expressed Fn14. TWEAK induced RANTES production, which was blocked with Fn14-Fc chimera (Figure 7b). We also found that TWEAK induced RANKL protein expression in human osteoblasts (Figure 7c).

These results suggest that TWEAK effects on osteoblasts may be relevant to the physiology of the bone.

## Discussion

To our knowledge, this is the first study that describes the effects of TWEAK on osteoblast-lineage cells. Previous studies have suggested that RANTES, derived from osteoblasts, is an important chemokine for the migration of osteoclasts [19] and that BMPs are important for osteoblast differentiation [15,16]. In addition, osteoblasts regulate osteoclast differentiation through RANKL expression [18]. Our results thus suggest that TWEAK may be a novel regulator for bone homeostasis through the modulation of osteoblast function and differentiation.

The current results clearly showed that TWEAK regulates RANTES production, BMP-2-induced differentiation, and RANKL expression in MC3T3-E1 cells in an Fn14-dependent manner. Polek *et al*. previously reported that TWEAK induced the differentiation of RAW264.7 monocyte/macrophage cells into osteoclasts in an Fn14-independent manner, suggesting that receptors other than Fn14 may exist on osteoclasts [12]. Therefore, it is still possible that there are some TWEAK-dependent, but Fn14-independent, responses in MC3T3-E1 cells.

We and others have reported that TWEAK/Fn14 interaction plays important roles in proliferation, migration, inflammatory responses, and death in a variety of cell types and that NF-κB and MAPK pathways principally mediate TWEAK/Fn14 biological activity [2,3,8-11]. Thus, to our knowledge, this is the first demonstration that TWEAK/Fn14 interaction also activates the PI3K-Akt pathway. De Ketelaere *et al*. reported that a short variant form of TWEAK (sTWEAK), unlike full-length TWEAK, was internalised in an Fn14-independent manner and colocalised with glycogen synthase kinase-3β (GSK-3β), one of the target molecules for the PI3K-Akt pathway in neuroblastoma cells [20]. Thus, it would be interesting to investigate the roles of GSK-3β in full-length TWEAK/Fn14-induced RANTES production in MC3T3-E1 cells.

It remains unclear how TWEAK inhibits BMP activities through the MAPK Erk pathway in MC3T3-E1 cells. BMP-Smad signaling is suggested to be involved in osteoblastic differentiation of MC3T3-E1 cells [21]. Previously, Kretzschmar *et al*. reported that the MAPK Erk pathway inhibited BMP-Smad signaling by inducing serine phosphorylation of the linker region of Smad proteins, resulting in the blockade of Smad nuclear translocation in a certain cell type [22]. It is thus possible that TWEAK interferes with the BMP-Smad pathway through activation of the MAPK Erk pathway in MC3T3-E1 cells. We are currently investigating this possibility.

We found significant TWEAK mRNA expression in MC3T3-E1 cells and RAW264 cells (Figure 6b). However, it is unlikely that TWEAK functions in an autocrine manner in MC3T3-E1 cells, because we did not observe any effects of Fn14-Fc chimera alone on RANTES production, BMP-2-induced differentiation, and RANKL expression. It should be determined in the future whether TWEAK is expressed in these cells at the protein level.

We found that TWEAK immunoreactivity was detected in morphologically osteoblast- or osteocyte-like cells, but not in chondrocytes, in the mouse bone tissue (Figure 6c). It appeared that TWEAK was detected mainly in the nucleus of the cells.

Although one report suggests the nuclear localisation of TWEAK in neuroblastoma cells [20], subcellular localisation of TWEAK in various cell types remains to be determined.

In summary, we showed that TWEAK/Fn14 interaction induced RANTES production through the PI3K-Akt pathway, inhibited BMP-2-induced differentiation through the MAPK Erk pathway, and upregulated RANKL expression through the MAPK Erk pathway in osteoblastic MC3T3-E1 cells. These results suggest a regulatory role of TWEAK/Fn14 interaction for osteoblast function. The findings that TWEAK was expressed in the healthy mouse bone tissue and that human osteoblasts expressed and responded to TWEAK also suggest that TWEAK may play an important role in bone physiology. Future studies should be aimed at further elucidating the *in vivo *roles of TWEAK/Fn14 interaction in healthy and pathological conditions of bone.

## Conclusion

TWEAK/Fn14 interaction induces RANTES production, inhibits BMP-2-induced differentiation, and upregulates RANKL expression in osteoblastic MC3T3-E1 cells. TWEAK/Fn14 interaction may thus play a role in osteoblast function and differentiation.

## Abbreviations

ALP = alkaline phosphatase; BMP = bone morphogenetic protein; ELISA = enzyme-linked immunosorbent assay; Fn14 = fibroblast growth factor-inducible 14; GAPDH = glyceraldehyde-3-phosphate dehydrogenase; GSK-3β = glycogen synthase kinase-3β; IgG = immunoglobulin G; mAb = monoclonal antibody; NF-κB = nuclear factor-κB; PBS = phosphate-buffered saline; PCR = polymerase chain reaction; RANKL = receptor activation of nuclear factor-κB ligand; RANTES = regulated upon activation, healthy T cell expressed and secreted; TGF = transforming growth factor; TNF = tumour necrosis factor; TRAF = tumour necrosis factor receptor-associated factor; TWEAK = tumour necrosis factor-like weak inducer of apoptosis.

## Competing interests

The authors declare that they have no competing interests.

## Authors' contributions

TA and JI were equally responsible for the experiments and data analysis and wrote the manuscript. MW, KH, YW, MS, KT, HO, and YH assisted in the experiments. HY and AN were responsible for the planning of the research and wrote the manuscript. All authors read and approved the final manuscript.
